# Energy Evolution and Fine Structure Effects in Typical Rocks Subjected to Impact Loading

**DOI:** 10.3390/ma19010003

**Published:** 2025-12-19

**Authors:** Ding Deng, Gaofeng Liu, Lianjun Guo, Yuling Li, Jiawei Hua

**Affiliations:** 1 School of Materials Science and Engineering, Shenyang University of Technology, Shenyang 110870, China; dingdeng@smail.sut.edu.cn; 2 School of Architecture and Civil Engineering, Shenyang University of Technology, Shenyang 110870, China; 164675825@smail.sut.edu.cn (G.L.); 15232768900@163.com (Y.L.); 15322939401@163.com (J.H.)

**Keywords:** impact load, energy evolution, mesoscopic structure, energy-time density

## Abstract

To investigate the mechanical behavior and energy evolution characteristics of various rock materials under impact loading, dynamic impact tests were conducted on five representative rock types using a split Hopkinson pressure bar (SHPB) apparatus, combined with X-ray diffraction (XRD) and scanning electron microscopy (SEM) techniques. The dynamic mechanical response, energy characteristics, mineral composition, and associated microstructural features of these typical rocks were systematically analyzed. The results show that basalt exhibits the highest peak strength, followed by blue sandstone and granite; all three display typical brittle failure characteristics, whereas red sandstone and green sandstone demonstrate greater ductility and plastic deformation capacity. By introducing the energy-time density index, the energy-time density of the rocks ranks from strongest to weakest as follows: green sandstone, red sandstone, granite, blue sandstone, and basalt. An innovative dynamic strength–energy-time density mapping model was established to elucidate the clustering and distinguishing characteristics of these rock materials. Assay results and mesoscopic images confirm the relationship between mineral composition and the fine structure of rock fragmentation mechanisms, highlighting that the critical transition from intergranular to transgranular fracture is the key mechanism governing impact pulverization. Furthermore, fractal analysis reveals that higher fractal dimensions are associated with more complex microcrack structures and may correlate with the corresponding energy dissipation intensity. These findings provide profound insight into the failure mechanisms of rocks under dynamic loading, offering significant theoretical value and engineering application prospects, particularly in fields such as mining excavation and rock mass stability assessment.

## 1. Introduction

In the domains of rock engineering and energy utilization, the mechanical response and energy evolution characteristics of rock materials subjected to impact loading represent a vital area of research. This is particularly pertinent in the contexts of mining, tunnel blasting, and underground engineering, where the high-strain-rate effect of impact loading significantly alters the mechanical behavior and damage modes of rocks. These alterations can, in turn, lead to dynamic failures such as rock bursts and cave-ins. Understanding the damage behavior of rocks under high-strain-rate conditions is thus crucial for maintaining rock stability and enhancing energy utilization.

The earliest relevant studies primarily focused on aerospace, defense applications, and ceramic matrix composites (CMCs). For instance, the impact between aircraft engine blades and casings involves complex energy transfer and dissipation processes that are strongly influenced by interfaces and interphase layers and are closely related to the Young’s modulus of the material [1]. Recent studies show that the application of non-oxide CMCs (e.g., SiC/SiC) in gas turbine blades has significantly improved their thermal and creep resistance while reducing maintenance requirements and extending service life through optimized interface design [2]. In the interaction between composite ceramic armor and projectile impact, the lightweight design of honeycomb ceramic structures enhances oblique penetration resistance, thereby increasing energy dissipation efficiency and improving overall impact toughness [3]. Further analysis reveals that the effect of interphase layer thickness in fiber-reinforced CMCs on matrix cracking is non-monotonic, and energy transfer processes can be optimized by adjusting interfacial friction and residual thermal stress. These research findings underscore the central role of interface engineering in enhancing the impact performance of CMCs and provide theoretical support for future material development [4,5].

Research on the mechanical behavior and energy dissipation characteristics of materials under impact loading is already extensive, particularly regarding the impact response of rocks, ceramics, and ceramic matrix composites (CMCs) [6]. The energy dissipation mechanisms of rocks under impact loading share certain similarities with those of ceramics and CMCs, all manifesting as crack initiation, propagation, and energy dissipation, leading to fragmentation under high-strain-rate conditions. However, significant differences exist between rocks and ceramic/CMC materials. As a natural polyphase material, rock exhibits greater anisotropy, complex pore structures, and heterogeneous mineral compositions, rendering its damage behavior considerably more complex [6]. Microcracks, grain boundaries, and variations in mineral composition within rocks significantly affect the modes of energy absorption and dissipation during impact. Therefore, investigating whether similar impact energy dissipation mechanisms apply to typical rocks under dynamic loading holds important academic and engineering significance.

Research indicates considerable variability in the mechanical behavior and energy evolution characteristics of different rock types under impact. Wang et al. [7] performed an experimental investigation into the acoustic emission and energy evolution characteristics of rocks under cyclic loading conditions, revealing that significant differences exist in the energy dissipation pathways and damage-critical points across various rocks during cumulative loading. They found that energy storage intensity is a key determinant of the damage mode exhibited by the rocks. Similarly, Meng et al. [8] developed a nonlinear evolution model of energy released by rocks during fracture through cyclic uniaxial loading tests on sandstone, limestone, and marble. Their findings highlighted essential differences in loading paths and energy dissipation modes among rocks with distinct lithologies. Li et al. [9] conducted repetitive impact loading tests on sandstone using the SHPB system, complemented by methods such as optical microscopy and nuclear magnetic resonance. Their results indicated that higher impact speed results in a denser internal crack formation within sandstone, enhancing its energy absorption rate. Notably, discrepancies were observed in the crack development mode and energy distribution mechanism among different lithologies. Additionally, Li et al. [10] investigated the impact degradation process of green sandstone, identifying three phases dominated by microcracks. They found that as the number of impacts increased, significant variations emerged in the rate of development and orientation of the fracture networks across various rock types, ultimately influencing their energy consumption during rupture.

The deformation and damage behavior of lithological materials at the macro scale fundamentally originates from the evolutionary processes governing their microstructure. Variations in mineral composition, particle structure, porosity, and fracture distribution result in distinct damage modes and energy response behaviors among different rock types under external loading conditions. It has been established that rock fragmentation under impact loading primarily arises from the synergistic interplay of intragranular and intergranular cracks, with the evolution path being influenced by factors such as the bonding strength between minerals, grain size, and the structural arrangement orientation [11]. Li et al. [10] focused on green sandstone, employing SEM and ultrasonic techniques to meticulously delineate the processes involved in microfracture initiation, propagation, and aggregation. Their findings highlighted that microfracture behavior plays a pivotal role in determining the macroscopic mechanical properties of the rock. Furthermore, the application of XRD technology has illuminated the principal influence of mineral composition across various lithological materials. For instance, rocks with a high quartz content are more susceptible to intracrystalline brittle fracture, whereas those composed of laminated structural minerals, such as mica analogues, exhibit a propensity for slippage and cleavage along intergranular interfaces [12]. Additionally, SEM technology provides direct visualization of the evolution of microcracks, transitioning from point and line defects to a network-like fracture system during loading, affirming that microcrack aggregation is a precursor to macroscopic damage [13].

In recent years, the integration of fractal theory into rock mechanics and rupture mechanisms studies has gained prevalence, particularly in elucidating the complex structures and energy dissipation capacities of rock rupture surfaces. This approach has demonstrated significant advantages. The fractal dimension, as a metric for quantitatively assessing geometric roughness and structural complexity, has become a critical index for characterizing the evolution processes of rupture surfaces [14].

Hu and Li [15] first applied fractal theory to studies on rock impact rupture, positing that a higher fractal dimension of the fractured section correlates with a more complex fracture network and enhanced energy dissipation intensity, serving as an effective factor for evaluating rocks’ impact resistance to failure. Zhang et al. [16] observed in their research on coal–rock composites that samples exhibiting higher fractal dimensions developed denser and multi-directionally expanding fracture systems, which demonstrated superior energy absorption and dissipation capabilities, thereby increasing the tendency for fracture and brittle destabilization within the rock body. Tian et al. [17], based on rock energy dissipation theory and fractal theory, analyzed the energy evolution characteristics and the fractal characteristics of fragmentation during the deformation and failure process of specimens. Luo and Gong [18] remarked on the clear coupling relationship between changes in fractal dimension and energy density responses during the fracturing process of red sandstone under contrasting loading rates, which can facilitate the identification of stages at which the rock approaches critical instability. Wang et al. [19] further identified that preloading stress has a moderating effect on the fractal dimension of rock during dynamic fracture processes, with the mechanism of influence manifesting through effects on crack generation density and penetration pathways. Moreover, Li et al. [20] examined the applicability of two fractal dimension calculation methods, CCM and SAM, through experimental analysis. By employing the enhanced SAM method to quantify the three-dimensional morphology of the fracture surface of sandstone, a significant correlation was identified between fractal dimension and rupture mechanisms, showing a decreasing trend in fractal dimension with the transition of damage mechanisms from tensile to shear fractures [20].

In the present study, five typical rock materials—red sandstone, green sandstone, blue sandstone, granite, and basalt—were selected as research objects. Dynamic impact mechanical tests were conducted on these rock materials, supplemented by XRD and SEM analyses for fine-scale observation. Innovatively integrating energy density and loading duration characteristics, an energy-time density index was introduced, and a dynamic strength–energy-time density model for the rocks was constructed; clustering analysis of lithology and energy characteristics was performed, effectively distinguishing differences in energy dissipation intensity and impact resistance among rock types. Furthermore, fractal dimension analysis was applied to fractured rock surfaces, serving as an index to precisely quantify the degree of microscopic fragmentation and providing new insights into the complexity of microcrack structures. The study further investigated the energy evolution in rocks under impact loading and its governing mechanisms, with the aim of elucidating how mineral composition and microstructure influence rock behavior under dynamic loading. This research is expected to provide a solid theoretical foundation for predicting rock mass stability and for the effective prevention and mitigation of related engineering disasters.

## 2. Materials and Methods

### 2.1. Specimen Selection and Processing

One of the main objectives of this study is to investigate the energy evolution and fine structure effects in typical rocks under impact loading conditions. This research involves conducting impact tests on representative rock samples, collecting stress–strain data throughout the impact process, and examining the rock composition as well as the fine structure of the fracture surfaces post-impact.

**Figure 1:**
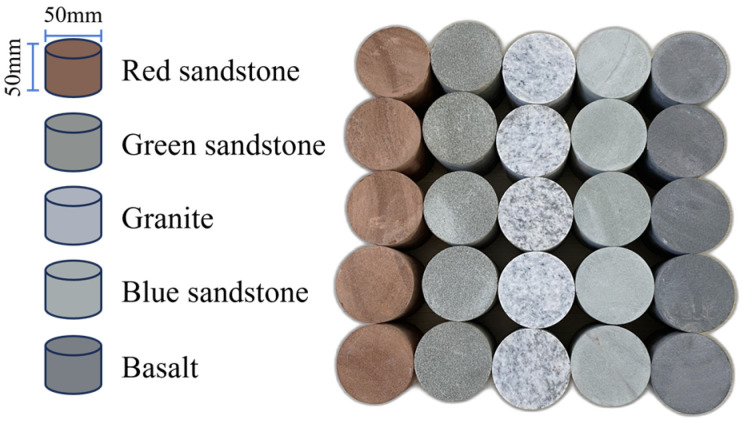
Standard cylindrical specimen prepared for tests.

To minimize the influence of inherent initial characteristics within individual rock specimens, all samples were collected from a uniform geographic area. The wet sampling method was then employed for specimen preparation, with standardized cylindrical specimens measuring 50 mm × 50 mm, as shown in Figure 1.

### 2.2. Test Setup and Methods

**Figure 2:**
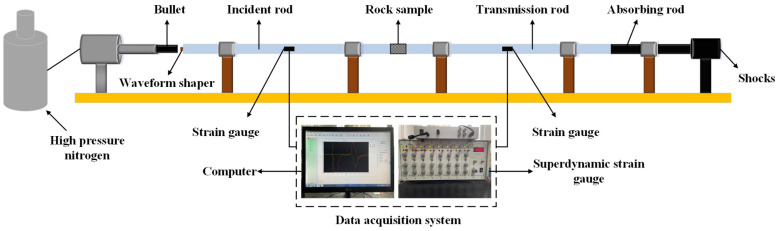
Schematic representation of the Split Hopkinson Pressure Bar (SHPB) experimental system.

In this experiment, the Split Hopkinson Pressure Bar (SHPB) system was used to carry out the study of dynamic mechanical properties of rock, and the test setup is shown in Figure 2. The system mainly consists of an impact bar, an incident bar, and a transmission bar, and the material used for the pressure bar is ultra-high-strength low-alloy steel, with a density of 7800 kg/m^3^ and a modulus of elasticity of 211 GPa. In order to improve the loading waveform and to promote the dynamic mechanical balance of the specimen, a piece of copper disc with a diameter of 10 mm and a thickness of 2 mm was pasted at the free end of the incident bar to serve as an impulse shaper. At the same time, a strain gauge was installed at the center of the incident and transmission rods to collect the strain signals propagated in the rods during the dynamic loading process.

The fundamental principle of X-ray diffraction involves utilizing the diffraction of X-rays by crystalline materials to ascertain their crystalline structure. X-ray diffraction (XRD) analyses were performed using a D/MAX-2600 X-ray diffractometer manufactured by Rigaku Company, Tokyo, Japan. This instrument employed copper (Cu) as the anode target material. Rock samples of various lithologies—including red sandstone, green sandstone, granite, blue sandstone, and basalt—were ground into dry powders with a particle size of less than 200 mesh. Each sample weighed no less than 0.5 g and was then subjected to XRD analysis to accurately obtain detailed microscopic information, including the mineral phase composition and degree of crystallinity. The continuous scanning mode facilitated the collection of characteristic data related to crystal structure and mineral composition by measuring the diffraction peaks of the samples, thereby providing a foundation for subsequent mineralogical analysis and structural characterization.

In this experiment, a MIRALMS field-emission scanning electron microscope (SEM) manufactured by TESCAN in the Brno, Czech Republic was utilized to methodically observe and analyze the micro-morphological characteristics of rock fractures. To comprehensively investigate the impact damage characteristics of rocks with varying lithologies under identical impact air pressure, the fractured samples of the damaged rocks were carefully cut into thin slices, each measuring approximately 10 mm^2^, and affixed to a specialized sample base for SEM analysis using conductive adhesive tape. Given that some rock samples exhibited magnetic properties, an ultrasonic oscillator with alcohol was employed to clean the samples prior to measurement. Furthermore, owing to the inherent low electrical conductivity of the rock, the surfaces of the samples were coated with a thin layer of gold under vacuum conditions before imaging. This treatment was essential to enhance surface conductivity and ensure clear, stable imaging during SEM observation. The SEM operated at an accelerating voltage of 20 kV to observe the microstructure of the samples.

### 2.3. Stress Equilibrium Verification

**Figure 3:**
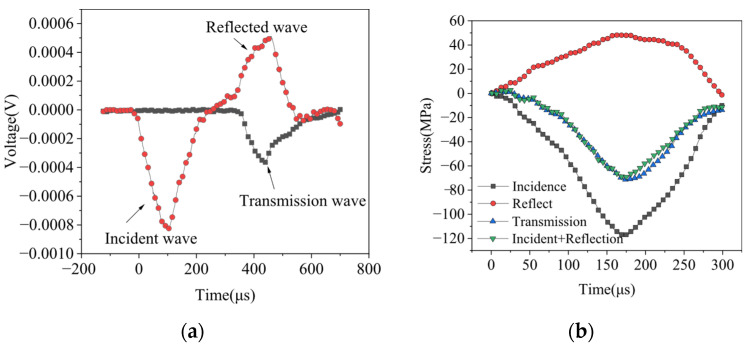
Typical stress wave signals and stress balance calibration curves: (**a**) typical voltage signals; (**b**) typical stress balance curves.

Determining whether the rock specimen attains a dynamic stress equilibrium prior to damage is critical for assessing the efficacy of the SHPB test. This test adheres to guidelines set forth by the International Society for Rock Mechanics (ISRM) and incorporates a copper sheet affixed to the end of the incident rod as a pulse shaper. This configuration extends the rise time of the loading waveform, effectively filtering high-frequency components and mitigating the effects of fluctuating dispersion. Such measures ensure a uniform distribution of stresses across both sides of the specimen throughout the loading process. Prior to the official test, a preliminary assessment was conducted to verify the stability of the equipment and to confirm stress balance during dynamic loading. Figure 3a illustrates the typical stress wave pattern observed in the rock specimen during loading, with incident, reflected, and transmitted waves exhibiting regular waveforms akin to sinusoidal shapes. This observation aligns with the fundamental requirements for achieving dynamic stress equilibrium in the specimen. Figure 3b presents a representative dynamic stress balance verification curve, where the superposition of the incident and reflected waveforms alongside the transmitted waveforms demonstrates a high level of consistency, further affirming that the testing apparatus effectively maintains stress balance at both ends of the specimen during the dynamic loading process. Consequently, this ensures the reliability and validity of the test results.

## 3. Results and Analysis

### 3.1. Dynamic Stress–Strain Curve

According to the three-wave method, the effective data collected are processed, and using the refined stress–strain signals, in conjunction with the one-dimensional stress wave theory applicable to elastic rods, the dynamic strength data for various rock types are calculated as follows [21,22,23]:

(1) $$\left\{\begin{array}{l} \sigma (t)=\frac{AE}{2A_{s}}\left[\varepsilon_{I}(t)+\varepsilon_{R}(t)+\varepsilon_{T}(t)\right]\\ \varepsilon (t)=\frac{C}{l_{s}}\int_{0}^{t}\left[\varepsilon_{I}(t)-\varepsilon_{R}(t)-\varepsilon_{T}(t)\right]dt\\ \dot{\varepsilon }(t)=\frac{C}{l_{s}}\left[\varepsilon_{I}(t)-\varepsilon_{R}(t)-\varepsilon_{T}(t)\right] \end{array}\right.$$

where *A*, *E*, and *C* in order are the cross-sectional area, elastic modulus, and longitudinal wave speed of the compression bar; *A_s_* and *l_s_* denote the cross-sectional area and length of the specimen, respectively; and *ε_I_*(*t*), *ε_R_*(*t*), and *ε_T_*(*t*) in order represent the incident strain, reflected strain, and transmitted strain of the compression bar.

Dynamic impact tests were conducted on the representative rock specimens using a split Hopkinson pressure bar (SHPB) apparatus at a uniform impact air pressure of 0.3 MPa. All tests were performed at room temperature. Five parallel tests were carried out for each rock type. Owing to the highly consistent basic characteristics of the test data, only one representative dataset and the corresponding failure pattern from each rock type were selected for comparative presentation and subsequent analysis of dynamic mechanical response and failure characteristics.

**Figure 4:**
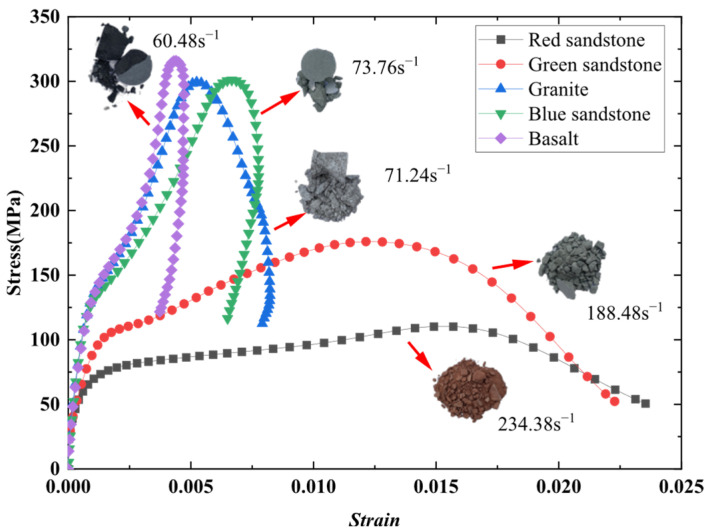
Stress–strain curves of five typical rocks.

The stress–strain curves of five typical rock specimens under the action of the same strain rate were obtained according to the calculation, as demonstrated in Figure 4.

Based on the stress–strain curves of five rock types (red sandstone, green sandstone, granite, blue sandstone, and basalt) illustrated in Figure 4, a marked difference in the mechanical response characteristics of these varied rock materials is evident. The maximum peak strength of basalt is recorded at 332.30 MPa, followed closely by blue sandstone and granite, with peak strengths of 306.04 MPa and 299.43 MPa, respectively. These three exhibit pronounced brittle failure characteristics, as indicated by a rapid decline in stress following the peak and a minimal strain range. This suggests a robust load-bearing capacity, albeit with limited ductility, which is characteristic of brittle rocks. In contrast, the peak strengths of green sandstone and red sandstone are lower, at 175.97 MPa and 116.85 MPa, respectively. The stress–strain curves for these materials do not demonstrate an immediate steep decline post-peak; instead, they reveal a gradual decrease, accompanied by a significantly larger strain range, indicating notable plastic deformation characteristics.

Initially, during loading, the stress–strain curve of each rock material presents a non-linear ascending phase. This is mainly attributed to the inherent non-homogeneity of rocks, which contain varying degrees of micro-cracks and voids that gradually close under applied loads. As stress reaches a specific threshold, the rocks transition into the elastic phase, exhibiting more pronounced elastic deformation characteristics. The slope of these curves during this phase can be utilized to ascertain the material’s elastic modulus.

It is noteworthy that in SHPB tests, two distinct failure modes are observed after the stress reaches its peak: (i) When the peak stress does not exceed the yield strength of the material, the specimen maintains fundamental integrity, primarily undergoing compaction of internal microcracks and pores. In the post-peak stage, the stored elastic strain energy is released, resulting in an unloading segment on the stress–strain curve. Owing to the generation and propagation of microcracks during loading, permanent deformation remains after complete unloading. During the loading–unloading cycle, energy dissipation occurs and forms a hysteresis loop (i.e., the stress rebound phenomenon); the area of the hysteresis loop determines the energy dissipation capacity of the material. (ii) When the peak stress exceeds the yield strength, the specimen undergoes irreversible failure, deformation accumulates progressively, and the stress–strain curve exhibits an open shape.

The main findings of this study can be summarized as follows: (i) Basalt and blue sandstone exhibit the highest peak strengths and follow the first failure mode, characterized by a stress rebound phenomenon and a rapid stress drop after the peak, which reflects typical brittle failure behavior. This suggests that, under high stress levels, the rocks store a large amount of elastic strain energy, which is rapidly released upon failure, leading to a relatively small ultimate strain. (ii) In contrast, although granite also conforms to the first failure mode, it exhibits a slight extension of the strain range, indicating a certain degree of plastic deformation; the stress still decreases rapidly after the peak, but the drop is less abrupt than that observed in basalt and blue sandstone. (iii) Green sandstone and red sandstone, on the other hand, exhibit the second failure mode without any stress rebound, and their peak strengths are further reduced. Their stress–strain curves display a gradually descending trend after the peak and are characterized by an open shape. Such behavior indicates that, after reaching the yield strength, red sandstone and green sandstone undergo irreversible plastic deformation; as loading continues, strain progressively accumulates, manifesting pronounced ductile characteristics together with a strong capacity for deformation accommodation.

### 3.2. Energy-Time Density Time-Course Curves

Based on the one-dimensional stress wave propagation theory, it is assumed that all kinetic energy imparted by the projectile launched by the pneumatic device is fully converted into the energy propagating into the bars, and energy loss at the contact interface between the specimen and the striker during testing is neglected. The incident energy is transmitted through the elastic bars to the specimen, where a portion is absorbed by the rock, while the remainder is reflected and transmitted. According to Equations (2)–(4), the incident wave energy (*E*_I_), reflected wave energy (*E*_R_), and transmitted wave energy (*E*_T_) can be calculated [24,25].

(2) $$E_{I}=A_{0}C_{0}E_{0}\int_{0}^{t}\sigma_{I}^{2}(t)dt$$

(3) $$E_{R}=A_{0}C_{0}E_{0}\int_{0}^{t}\sigma_{R}^{2}(t)dt$$

(4) $$E_{T}=A_{0}C_{0}E_{0}\int_{0}^{t}\sigma_{T}^{2}(t)dt$$

where *E*_I_, *E*_R_, and *E*_T_ represent the incident energy, reflected energy, and transmitted energy, respectively. *σ*_I_, *σ*_R_, and *σ*_T_ denote the incident stress, reflected stress, and transmitted stress, respectively.

In SHPB tests, the energy absorbed by the rock can be expressed as the incident energy minus the reflected and transmitted energies. The absorbed energy includes the dissipation energy required for internal crack propagation and the formation of new fracture surfaces, the kinetic energy of ejected fragments, and other forms of dissipated energy. In impact testing, other dissipative terms are generally neglected, and according to the findings of reference [26], the kinetic energy of ejected fragments accounts for approximately 5.05% of the total absorbed energy. Therefore, it is reasonable to approximate that the energy absorbed by the rock is primarily equivalent to the dissipation energy associated with internal crack propagation.

In simplified terms, during impact testing, the energy absorbed by the rock is obtained from Hopkinson test calculations, while the kinetic energy of ejected fragments and other dissipative energies are neglected, so the absorbed energy is approximated as the dissipation energy during the rock failure process. Therefore, in the subsequent analysis and discussion, the term “dissipated energy” is used throughout.

The dissipated energy (*E*_D_) of a rock specimen under dynamic loading can be calculated using Equation (5).

(5) $$E_{D}=E_{I}-E_{R}-E_{T}$$

To minimize the influence of specimen volume, the dissipated energy per unit volume is defined as the dissipated energy density according to Equation (6) [27]. Energy density reflects the dissipation energy during the rock fracture process, rather than the elastic energy stored prior to failure.

(6) $$U_{d}=\frac{E_{D}}{V}$$

where *U_d_* represents the energy density, *E_D_* denotes the absorbed energy, and *V* is the volume.

In the current investigation, we propose a specific index, Energy-Time Density (*E_VT_*), which considers both energy intensity and action time. Energy-time density reflects the intensity of dissipated energy per unit volume of rock per unit time.

Based on the stress–strain curve data, the energy-time density of the rock (*E_VT_*) can be further calculated as:

(7) $$E_{VT}=\frac{U_{d}}{T}$$

where *E_VT_* represents the energy-time density, *U_d_* denotes the energy consumption density, and *T* is the reflected wave action time.

**Figure 5:**
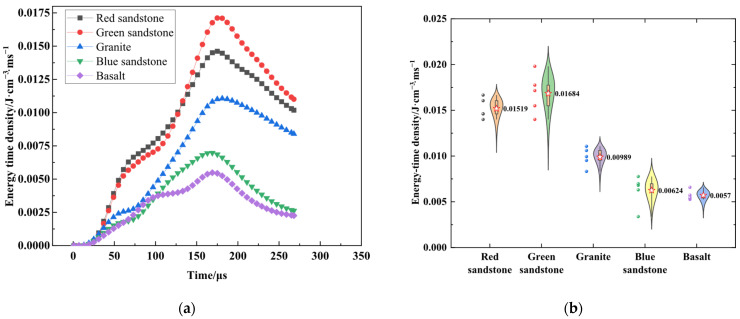
Plots of the energy-time density data for a typical rock: (**a**) energy-time density time-course curves for typical rocks; (**b**) plot of peak energy-time density for a typical rock.

The energy-time density data of typical rocks is shown in Figure 5. Figure 5a illustrates the time course curves of energy-time density for various lithologies, including red sandstone, green sandstone, granite, blue sandstone, and basalt, under uniform shock air pressure conditions. The energy-time density profiles for all rock types exhibit a rapid ascent to a peak value, followed by a gradual decline. Among these, green sandstone displays the highest peak energy-time density at 0.01715 J·cm^−3^·ms^−1^, followed closely by red sandstone at 0.01462 J·cm^−3^·ms^−1^. The peaks for granite and blue sandstone are comparatively moderate, while basalt demonstrates the lowest peak density. This indicates that variations in lithology have a significant impact on the peak energy-time density and the dynamic response characteristics under identical shock air pressure conditions.

The violin plots in Figure 5b present a statistical analysis of the distribution characteristics of peak energy-time density obtained from repeated tests on the five rock types. Significant differences in peak energy-time density are observed among the different lithologies, and the degree of data dispersion varies within each rock type. Green sandstone exhibits the widest data distribution range, indicating greater dispersion in its peak energy-time density values; concurrently, its higher peak values demonstrate the strongest overall energy dissipation intensity. In contrast, red sandstone, granite, and blue sandstone display more concentrated data with narrower distribution ranges, reflecting relatively stable peak energy-time densities. Conversely, basalt shows lower overall peak values with moderate dispersion, indicating the weakest energy dissipation intensity.

Utilizing the energy-time density and dynamic strength data of the rocks under consistent loading, a scatter plot depicting the dynamic strength–energy-time density distribution is constructed, with dynamic strength on the horizontal axis and peak energy-time density on the vertical axis. Additionally, a two-dimensional probability density map is generated via kernel density estimation to evaluate the probability density of the data points [28]:

(8) $$f(x,y)=\frac{1}{nh_{x}h_{y}}\sum_{i=1}^{n}K\left(\frac{x-x_{i}}{h_{x}},\frac{y-y_{i}}{h_{y}}\right)$$

where *f*(*x*, *y*) represents the probability density function, *n* denotes the number of data points, *h_x_* and *h_y_* are the bandwidth parameters, and *K* is the kernel function, commonly taken as a Gaussian type.

**Figure 6:**
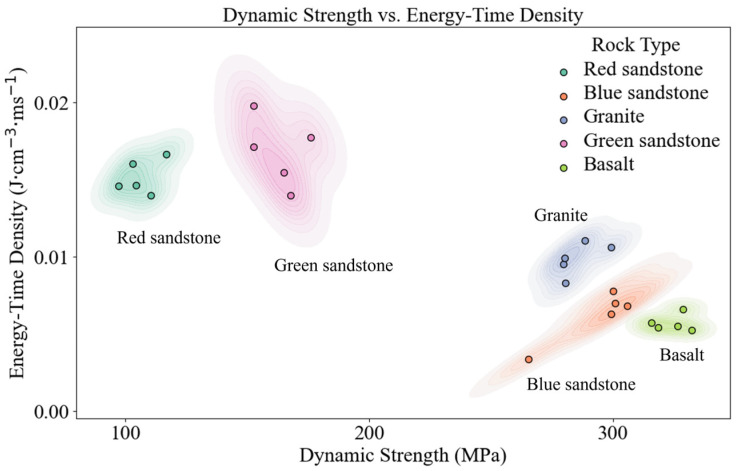
Model plot of the dynamic strength–energy-time density peak distribution.

The two-dimensional kernel density estimates of peak dynamic strength and energy-time density for various typical rock types under the same impact air pressure conditions are illustrated in Figure 6. Analysis of the scattering patterns and kernel density distributions reveals pronounced clustering characteristics and distinct differences in the dynamic strength and energy-time density across the lithologies studied.

Red sandstone and green sandstone are observed to manifest higher energy-time densities coupled with lower dynamic strengths, with their clustering areas positioned relatively close to one another. The translation is: this indicates that under impact loading, these two rock types have higher energy dissipation intensities and are more prone to fracture.

In contrast, granite, basalt, and blue sandstone exhibit the dual attributes of high dynamic strength alongside low energy-time density; however, the clustering areas for these three lithologies remain relatively distinct.

With respect to impact resistance, the performance ranks as granite > green sandstone > basalt; all three rock types exhibit relatively strong impact resistance. However, in terms of energy dissipation intensity, the order is reversed: basalt > green sandstone > granite.

### 3.3. Analysis of Fine-Scale Structural Effects

#### 3.3.1. XRD Diffraction Test

Rocks, as quintessential products of natural geological processes, can be categorized into three primary groups, magmatic rocks, sedimentary rocks, and metamorphic rocks, based on their genesis and inherent properties. Prolonged geological actions result in notable variations in mineral composition and alterations in the structural relationships among mineral particles across different rock types. These microstructural characteristics represent critical factors influencing the energy evolution and dissipation behavior of rocks [29]. To elucidate the impact of lithological variations on the dynamic mechanical properties of rocks, this study systematically examines the mineral compositions and degrees of mineral crystallization in specimens representing various lithologies, utilizing X-ray diffraction (XRD) technology.

**Figure 7:**
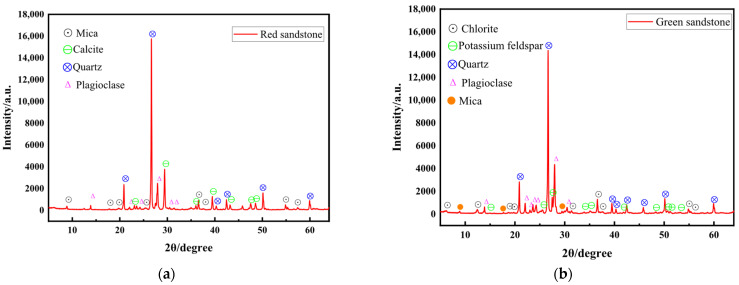

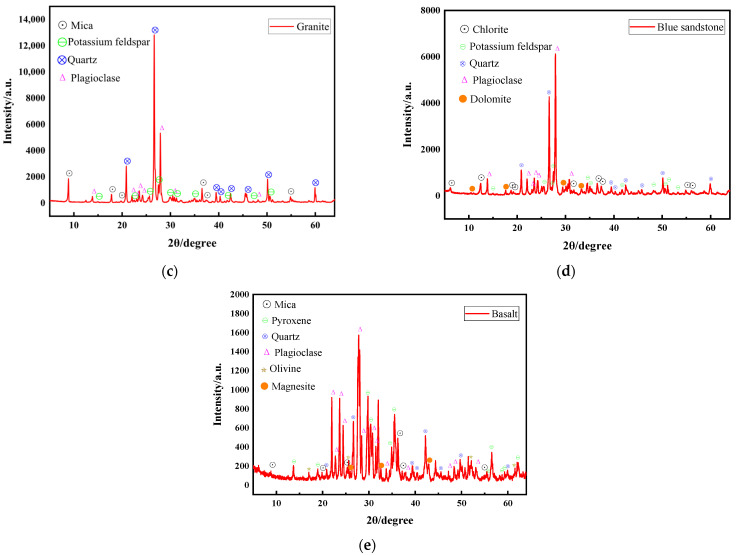
Plots of the XRD data of a typical rock: (**a**) red sandstone; (**b**) green sandstone; (**c**) granite; (**d**) blue sandstone; (**e**) basalt.

**Figure 8:**
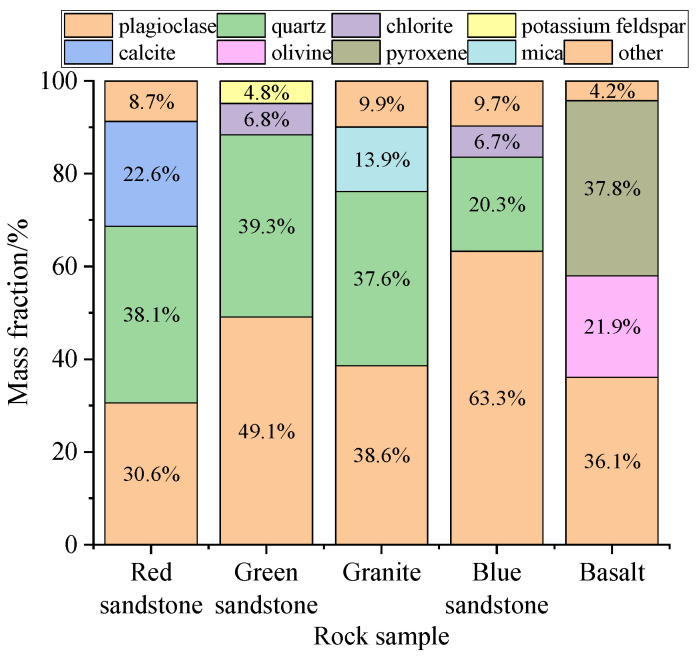
Main mineral mass distribution of each rock sample.

The X-ray diffraction (XRD) data of typical rocks and the main mineral mass distribution of each rock sample are shown in Figure 7 and Figure 8. Red sandstone is primarily composed of quartz (38.1%), plagioclase feldspar (30.6%), and calcite (22.6%). In contrast, green sandstone is dominated by plagioclase feldspar (49.1%) and quartz (39.3%). Blue sandstone mainly contains plagioclase feldspar (63.3%) and quartz (20.3%). The relatively narrow diffraction peaks of these three rock types indicate high crystallinity of their mineral components, revealing pronounced brittle characteristics. Further peak density analysis shows that red sandstone and green sandstone exhibit higher energy dissipation characteristics, whereas blue sandstone, owing to its enrichment in minerals such as dolomite and chlorite, displays lower energy dissipation and consequently enhanced ductility.

The primary mineral composition of granite includes plagioclase feldspar (38.6%), quartz (37.6%), and mica (13.9%). Its narrow diffraction peaks indicate high mineral crystallinity, manifesting pronounced brittleness. At the same time, the presence of mica enhances its toughness. Combined with data on the temporal variation in peak density, the energy dissipation intensity of granite is lower than that of red sandstone and green sandstone.

The mineral composition of basalt is dominated by pyroxene (37.8%), plagioclase feldspar (36.1%), and olivine (21.9%). The diffraction pattern of basalt is relatively complex, with broader diffraction peaks than other rock types, reflecting lower mineral crystallinity. Nevertheless, owing to the abundance of brittle minerals such as pyroxene, feldspar, and olivine, basalt exhibits pronounced brittleness and relatively low energy dissipation.

The primary mechanical properties of rocks, including brittleness, toughness, and strength, are predominantly governed by their mineral composition. In accordance with previous studies [30,31,32,33,34], rocks containing high proportions of quartz and feldspar exhibit pronounced strength and brittleness, resulting in a rigid structure that is prone to brittle failure. Conversely, rocks enriched in calcite typically display reduced brittleness and enhanced ductility, permitting a certain degree of plastic deformation. An increase in the content of platy soft minerals (such as mica) markedly reduces rock strength while simultaneously enhancing toughness and anisotropy. Furthermore, rocks enriched in olivine and pyroxene generally exhibit exceptionally high hardness and brittleness.

#### 3.3.2. Electron Microscope Scanning Image

Five representative rock types—red sandstone, green sandstone, granite, blue sandstone, and basalt—were selected for this study. The micro-morphology of the fracture surfaces induced by impact loading was methodically observed and analyzed to elucidate the distinct characteristics of the rock fracture mechanisms and micro-damage modes.

The microscopic morphology of rock fractures can be categorized into two primary types: tensile and shear fractures. The microscopic characteristics of tensile fractures primarily manifest as deconvolutional fractures and along-crystal fractures, often accompanied by a large number of microcracks. The diversity of tensile fracture morphologies encompasses various forms, including step-like patterns, river-like patterns, plate-like patterns, and icing sugar-like patterns, among others, totaling nine distinct forms. In contrast, shear fractures can be further subdivided into slip fractures and microporous aggregation fractures, with their typical microscopic morphologies comprising eight types, such as serpentine sliding patterns, parallel sliding line patterns, and stripe patterns [35,36].

**Figure 9:**
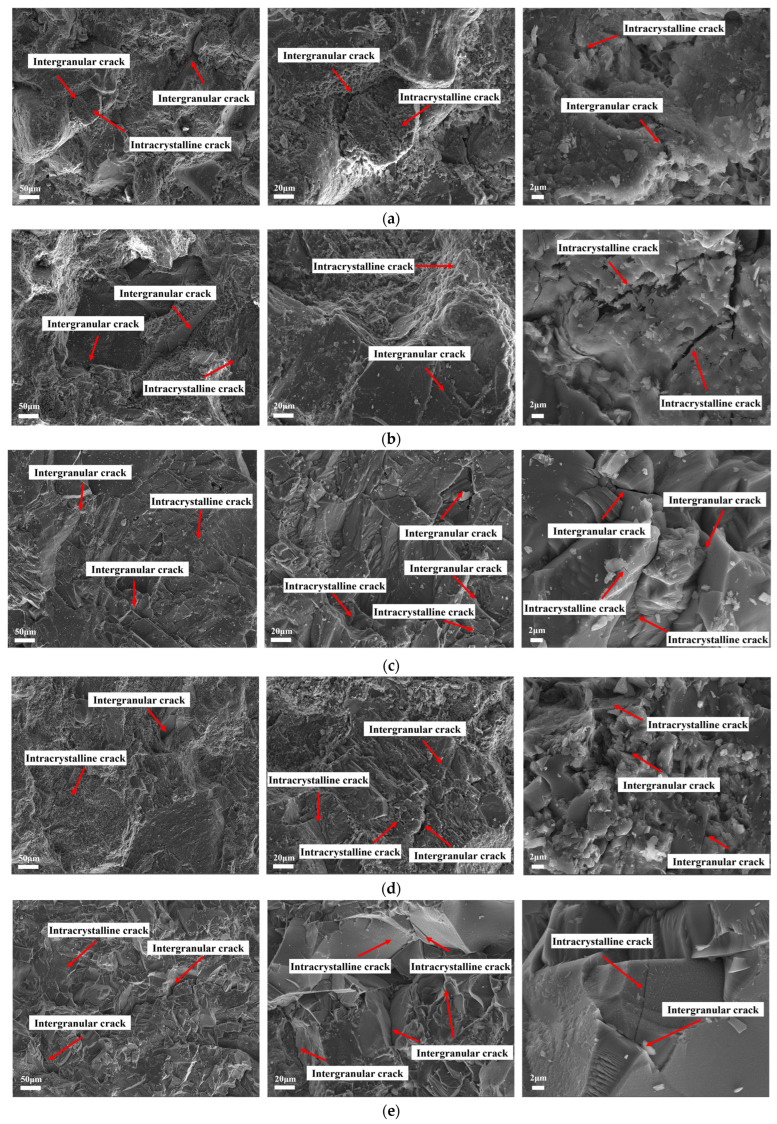
SEM scan of a typical rock: (**a**) red sandstone, stepped; (**b**) green sandstone, stepped; (**c**) granite, slate; (**d**) blue sandstone, parallel slip; (**e**) basalt, shear slip.

The electron microscope scanning tests conducted on five representative rocks under controlled impact air pressure reveal significant insights into their microstructures, as shown in Figure 9. The microstructures of red sandstone and green sandstone exhibit pronounced perforation cracks, robust crack penetration, and weak intergranular cementation, presenting a step-like morphology. The predominant mode of damage in these rocks is mainly characterized as tensile damage, leading to macroscopic crushing features. In contrast, granite displays well-defined crystal grains and a dense structure; crack propagation in granite is chiefly affected by both crystal penetration and along-crystal fracture, resulting in pronounced brittleness. The microscopic morphology of granite is mainly characterized by plate and granular features, with macroscopic damage reflecting jaw damage, which manifests as rupture surfaces and the formation of partially pulverized granular features.

The microscopic fracture characteristics of blue sandstone and basalt reveal the simultaneous occurrence of through-crystal and along-crystal fractures, with noticeable local crushing of crystal grains. However, when compared to red sandstone and green sandstone, the degree of aggregation in the crystal-penetrating fractures is comparatively lower. The microscopic morphology in these rocks is predominantly characterized by shear fracture features, such as serpentine sliding patterns and parallel slip line patterns, leading to macroscopic results that include the formation of localized shear zones around the fracture surfaces and the development of shear damage surfaces, with flaky fragments evident.

In summary, the rock crushing process is influenced by both intracrystalline and intergranular fractures; however, the transition from intergranular rupture to intracrystalline rupture at the microscale is fundamentally responsible for impact pulverization and the resultant crushing behavior.

#### 3.3.3. Fractal Characterization of the Broken Section

According to existing investigations, the fractal dimension of rock breakage remains identical regardless of sample size. Whether analyzing a small sample of 10 mm or a larger specimen measuring 400 mm, and extending even to fault lines spanning thousands of kilometers, the fractal dimension values exhibit striking similarity. This consistency highlights the self-similar structure of rock breakage, which can be characterized as geometrically fractal [37]. Consequently, the SEM images of the fracture surfaces of typical rock specimens could undergo fractal analysis, revealing analogous patterns in the variation in fractal dimensions of the broken surfaces under the influence of air pressure.

In this study, the fractal dimension of the fractured sections of rock samples was determined utilizing the box-counting method for fractal analysis. The computed box dimension effectively captures the geometric complexity inherent in the fractured surfaces. [38]:

(9) $$d_{\varepsilon }=\underset{k\to \infty }{\lim}\frac{\ln N_{\delta_{k}}(F)}{-\ln\delta_{k}}$$

where $\delta_{k}$ denotes the edge length of the mesh and $N_{\delta_{k}}$ represents the number of meshes intersecting the fractal set *F*.

**Figure 10:**
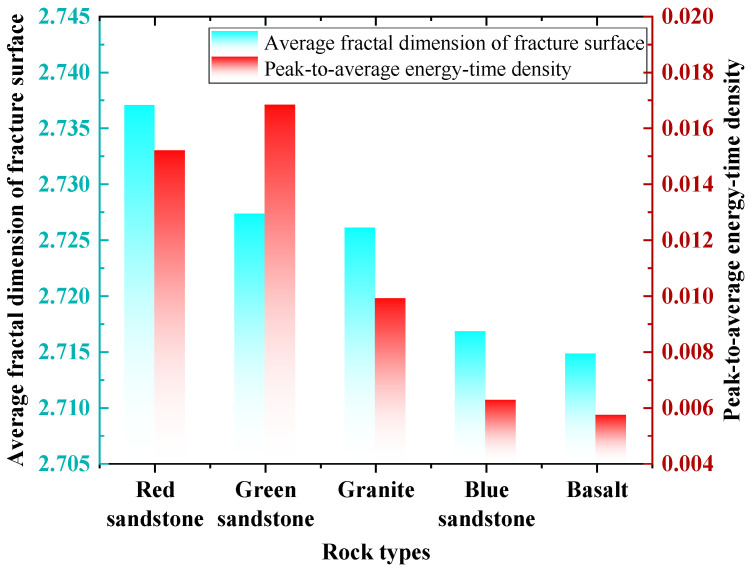
Plot of the fractal dimensions of broken section in terms of the energy-time density data for a typical rock.

Figure 10 presents the results of peak fractal dimension and energy-time density obtained from SEM image analysis of the fracture surfaces of various rock types under identical impact air pressure conditions. The analysis reveals significant differences in both fractal dimension and energy-time density among the different rock types. Specifically, fractal dimension measurements indicate that red sandstone, green sandstone, and granite exhibit higher values, suggesting that these rocks are more prone to developing complex microcrack structures during fragmentation. In contrast, blue sandstone and basalt show slightly lower fractal dimensions, implying that their fracture surfaces are relatively more uniform compared to the other rock types. Furthermore, evaluation of the peak energy-time density data shows that green sandstone possesses the highest value, indicating its superior energy dissipation intensity. The energy dissipation intensity of the rock types ranks in descending order as: green sandstone, red sandstone, granite, blue sandstone, and basalt.

In conjunction with previous studies, Ma et al. [39] conducted dynamic impact tests and, by combining fractal dimensions of fragment mass distribution with energy characteristics, found that the greater the energy absorbed by the rock, the more pronounced its fractal characteristics become. Pan et al. [40] systematically integrated fractal characteristics of fracture surfaces with energy features, revealing a structural relationship between failure mechanisms and energy evolution. Analysis of the experimental data indicates that, although the patterns observed in the figures suggest a potential correlation between fractal dimension and energy dissipation, the limited number of current SEM microscopic observation samples precludes rigorous statistical validation of this correlation using methods such as analysis of variance (ANOVA). To definitively establish a quantitative relationship between fractal dimension and energy dissipation intensity, more detailed analyses and additional data—such as microstructural investigations or tests under varying conditions—are required to confirm the existence of such a correlation. These future studies will further elucidate the intrinsic failure mechanisms of rocks under high-strain-rate loading and provide a more reliable theoretical foundation for engineering blasting, rock mass stability assessment, and protective design.

## 4. Conclusions

In this study, dynamic impact tests were conducted on five representative rock types using a split Hopkinson pressure bar (SHPB) system. Combined with X-ray diffraction (XRD) and scanning electron microscopy (SEM) techniques, the mechanical response and energy evolution characteristics of these rocks under impact loading were systematically analyzed. An innovative index termed energy-time density was introduced, and a dynamic strength–energy-time density mapping model was established. Mineral composition and fracture mechanisms of the typical rocks were investigated at the mesoscopic scale. Through quantitative and qualitative analyses, the following principal conclusions were drawn:

(1) The mechanical properties of the typical rocks exhibit significant differences. Basalt displays the highest peak strength of 332.30 MPa, followed by blue sandstone and granite; these three rock types predominantly exhibit brittle failure. In contrast, green sandstone and red sandstone demonstrate stronger plastic deformation capacity with lower peak strengths, and their failure modes are dominated by ductile fracture.
(2) The energy-time density varies markedly among the different rocks. Green sandstone exhibits the highest energy dissipation intensity, with a peak energy-time density of 0.01715 J·cm−3·ms−1. The energy dissipation intensities of red sandstone, granite, and blue sandstone decrease progressively, whereas basalt displays the lowest energy-time density.
(3) The dynamic intensity and energy-time density mapping model effectively illustrates the clustering characteristics and distinctions among rock materials. Notably, red sandstone and green sandstone tend toward high energy dissipation intensities coupled with low dynamic strength, whereas granite, blue sandstone, and basalt exhibit high dynamic strength accompanied by low energy dissipation intensities, demonstrating a completely inverse relationship between energy-time density and dynamic mechanical properties.
(4) The mineral composition and microstructure of rocks play a pivotal role in influencing their crushing mechanisms. The crushing processes of these rocks primarily involve intracrystalline fracture and intergranular fracture; however, the conversion of intergranular fractures to intracrystalline fractures at a finer scale is identified as the fundamental cause of impact crushing.
(5) A higher fractal dimension indicates that the rock is more prone to forming complex microcrack structures during the fracturing process. A certain correlation may exist between fractal dimension and energy dissipation; however, owing to the limitations of the current SEM dataset, this relationship cannot be statistically confirmed using analysis of variance (ANOVA). Additional microstructural analysis data or further tests under varying experimental conditions are required to validate this potential relationship.

## Data Availability

The data presented in this study are available on request from the corresponding author due to institutional confidentiality agreements and data privacy considerations.
